# An unusual presentation of ossified spinal meningioma: case report and literature review

**DOI:** 10.3389/fonc.2023.1259508

**Published:** 2023-09-27

**Authors:** Wen-Bin Xu, Nai-Kun Sun, Di-Xin Cai, Ding-Qiang Chen, Yuan Niu, Fang Jia, Guang-Xun Lin, Gang Rui

**Affiliations:** ^1^ Department of Orthopedics, The First Affiliated Hospital of Xiamen University, School of Medicine, Xiamen University, Xiamen, China; ^2^ The Third Clinical Medical College, Fujian Medical University, Fuzhou, China; ^3^ Department of Orthopedics, The First Affiliated Hospital of Nanchang University, Nanchang, China

**Keywords:** dorsal completely ossified meningioma, spinal meningioma, ossification, thoracic spine tumor, literature review

## Abstract

**Background:**

Spinal meningioma is a common intraspinal tumor, which mainly occurs in the thoracic spine. Ossified meningioma (OSM) is an extremely rare histological variant. Our article reports a rare patient with dorsal complete OSM and reviews this subject.

**Case presentation:**

A 68-year-old woman presented with a one-year history of progressive weakness in both lower limbs with gait disturbance. Physical examination revealed hypoesthesia with a sensory level below T10. Babinski and pathological signs on both sides were weakly positive. Magnetic resonance imaging (MRI) showed a mass at the T10 to T11 level causing severe compression of the spinal cord. Computed tomography (CT) showed complete ossification of the mass. 18F-Fluoro-deoxy-glucose positron emission tomography CT (18F-FDG PET/CT) scan combined with MRI revealed that the mass was an intradural extramedullary high-density ossified nodule. The patient underwent a gross total resection of the mass and pathologic examination indicated that the mass was a meningioma with diffused psammomatous bodies.

**Conclusion:**

We identified a rare case of dorsal complete OSM occurring in a 68-year-old woman. After complete surgical resection, although there were complications such as cerebral fluid leakage and fever, the patient finally recovered with a satisfactory result.

## 1 Introduction

Spinal meningioma accounts for about one-quarter of total primary intraspinal tumors, which is the second commonest intraspinal tumor following neurilemmoma (1). Spinal meningioma can occur in the extramedullary intradural and epidural areas, but the vast majority are intradural tumors (2). Although spinal meningioma is common clinically, calcification or ossification of spinal meningioma is uncommon, accounting for 1% to 5% of all spinal meningioma (3). Complete ossified meningioma (OSM) is extremely rare and is considered to be a rare histological variant. At present, spinal meningioma is not sensitive to radiotherapy and chemotherapy, and safe gross total resection remains the standard of care (3).

Here, a case of dorsal complete OSM is reported and the relevant subject is reviewed.

## 2 Case report

A 68-year-old woman presented with a one-year history of progressive weakness in both lower limbs with gait disturbance. Physical examination revealed normal muscle force of both lower limbs and hypoesthesia with a sensory level below T10. Babinski and pathological signs on both sides were weakly positive.

**Figure 1:**
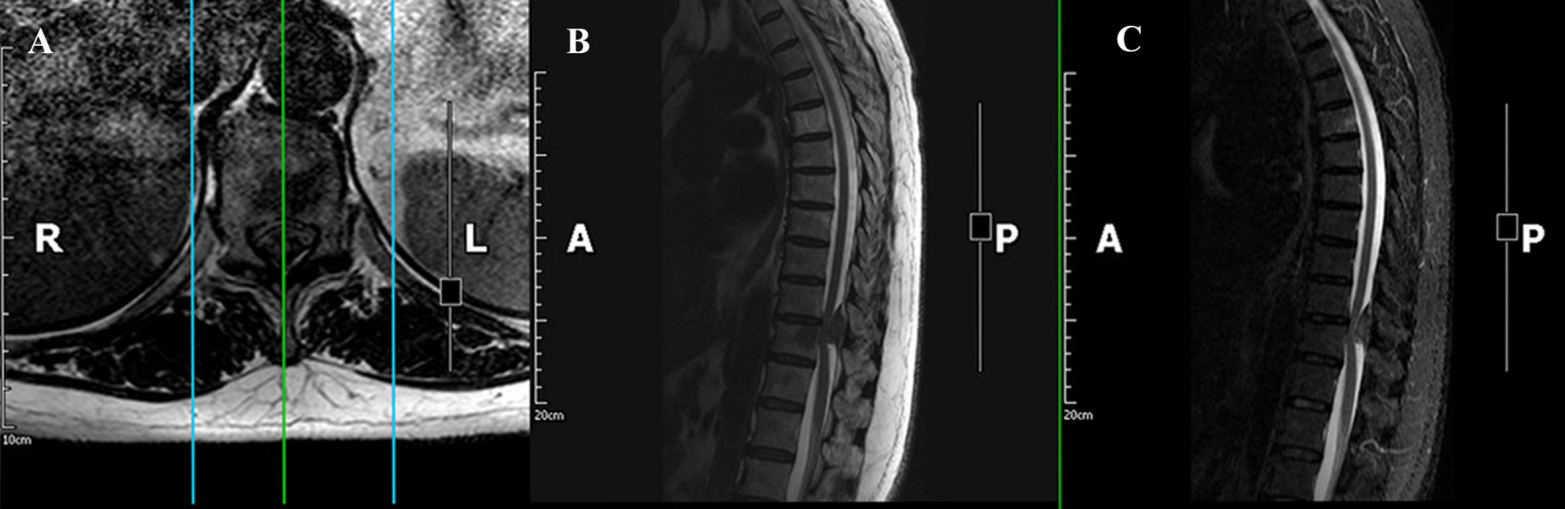
MRI demonstrated a mass between the T10 and T11 levels. **(A)** Axial T2-weighted image; **(B)** Sagittal T2-weighted image; **(C)** Sagittal T2-weighted image with fat-suppression.

**Figure 2:**
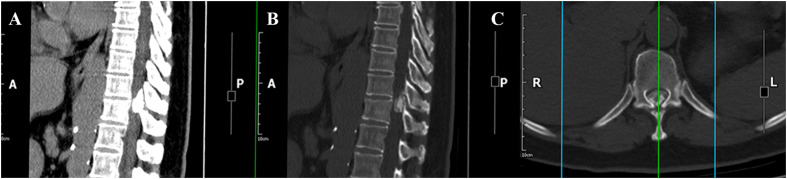
CT revealed complete ossification of the mass. **(A)** Sagittal CT image on bone window; **(B)** Sagittal CT image on soft-tissue window; **(C)** Axial CT image.

**Figure 3:**
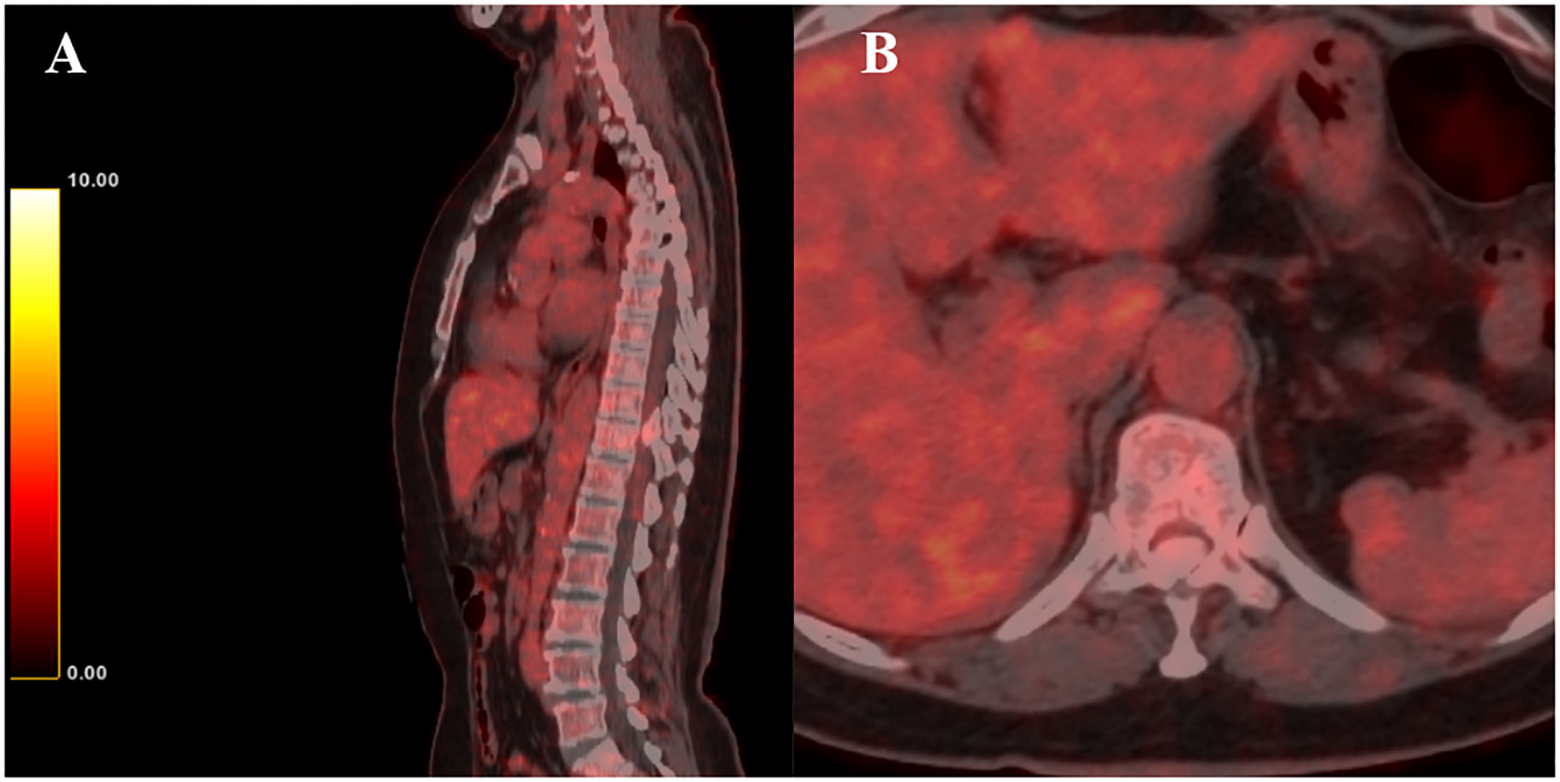
18F-FDG PET/CT revealed that the mass was an intradural extramedullary high-density ossified nodule. **(A)** Sagittal 18F-FDG PET/CT image; **(B)** Axial 18F-FDG PET/CT image.

Magnetic resonance imaging (MRI) demonstrated a mass between the T10 and T11 levels causing severe compression of the spinal cord (**Figure 1**). Computed tomography (CT) scan revealed complete ossification of the mass (**Figure 2**). 18F-Fluoro-deoxy-glucose positron emission tomography CT (18F-FDG PET/CT) scan combined with MRI revealed that the mass was an intradural extramedullary high-density ossified nodule (spinal meningioma)? (**Figure 3**).

**Figure 4:**
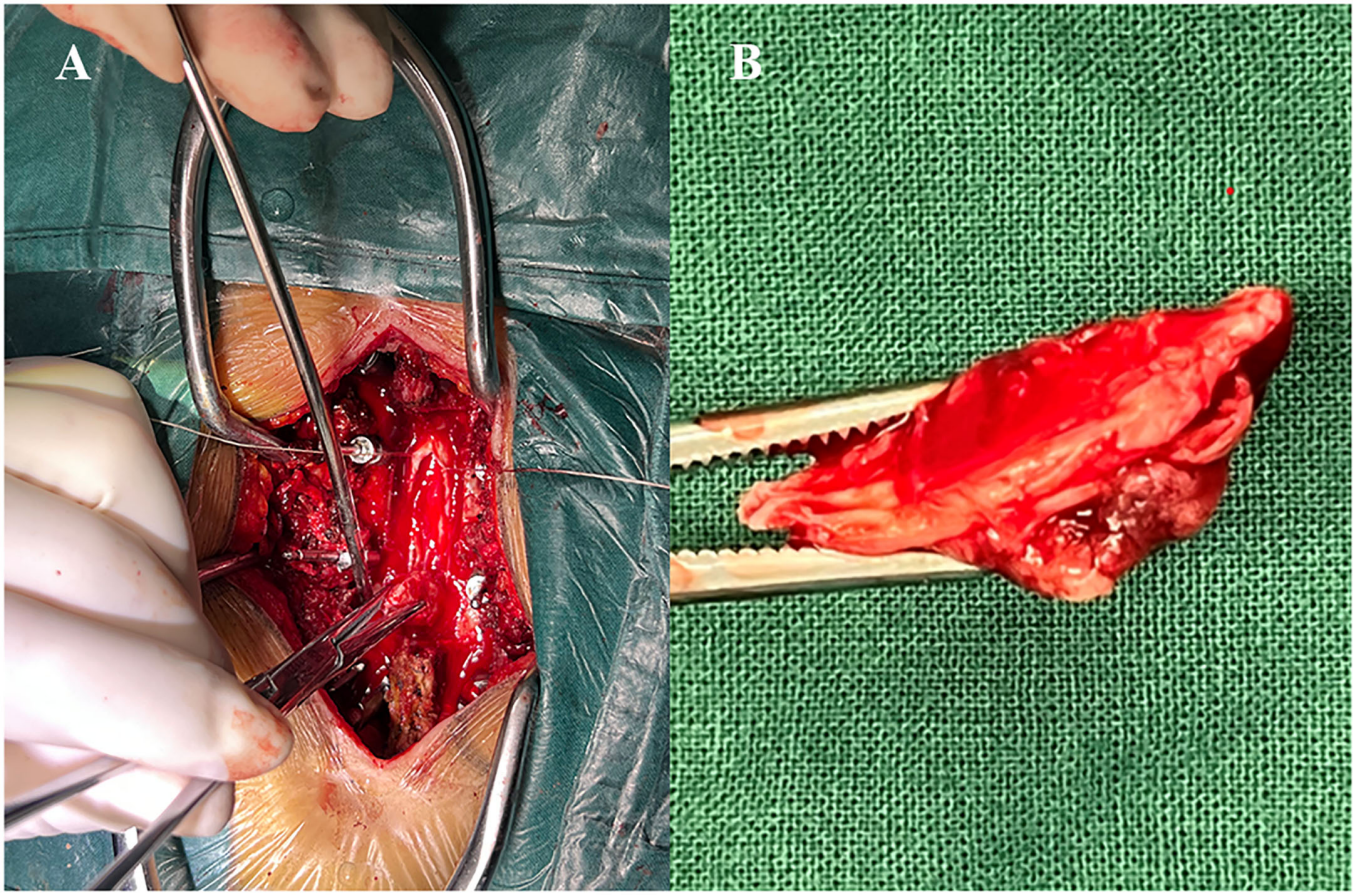
**(A)** Intraoperatively the mass was exposed by a dura incision; **(B)** The mass was completely resected.

**Figure 5:**
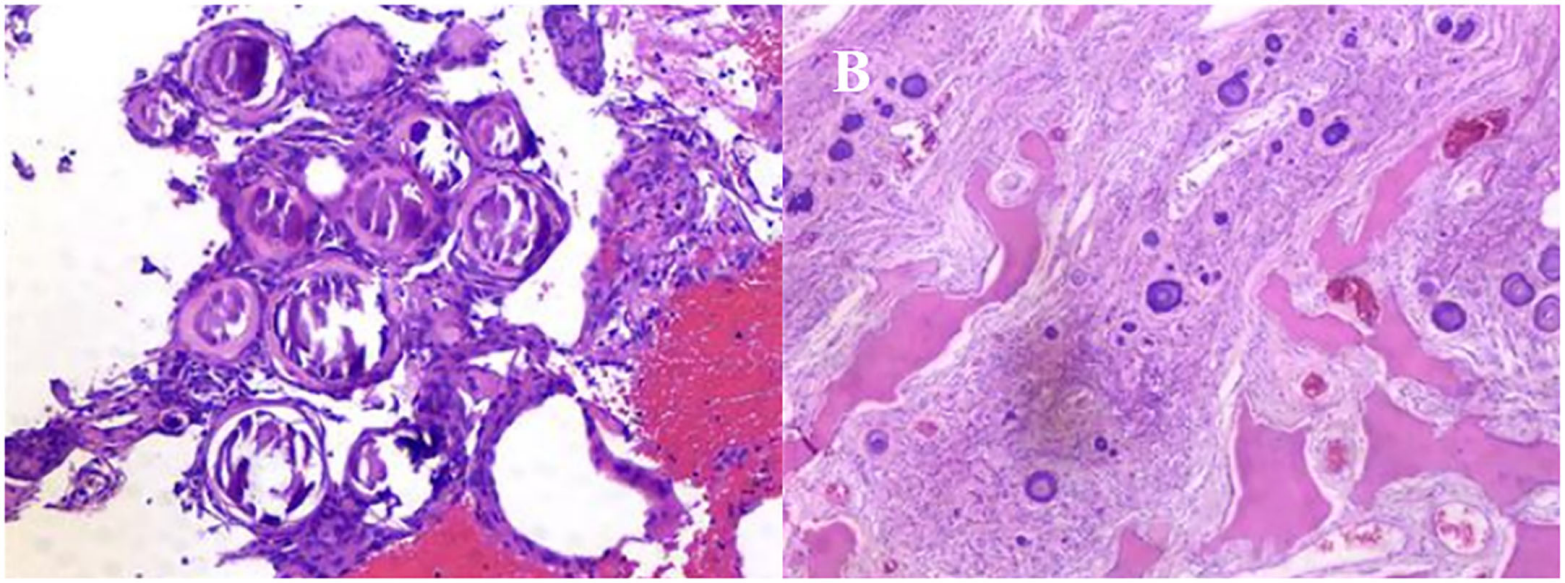
Histopathological examination of the resected tissue revealed that the mass was a meningioma with diffused psammomatous bodies.

Under general anesthesia, T9 to T12 transpedicular screw fixation was performed. Subsequently, the complete T10 to T11 spinous process and bilateral lamina of vertebra, and part of the spinous process and bilateral lamina of vertebra at the lower margin of T9 were removed under intraoperative neuromonitoring. Dorsal complete ossification of the intradural mass was revealed after full exposure of the dura of the corresponding segment (**Figure 4**). Then, the dura was cut along the edge of the mass and the mass was found to be heavily adhered to the arachnoid and spinal cord. After the careful separation between the tumor base and the adherent arachnoid, the mass was removed intact and then the dura mater was closed with a dural patch. Histopathological examination of the resected tissue revealed that the mass was a meningioma with diffused psammomatous bodies (**Figure 5**).

**Figure 6:**
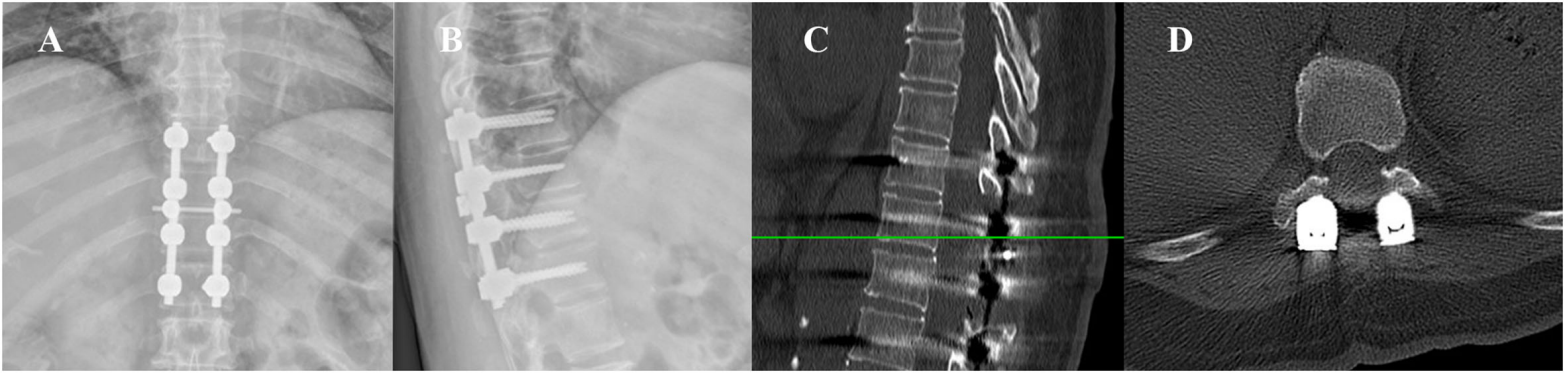
The postoperative examination of the patient. **(A, B)** DR revealed appropriate internal fixation; **(C, D)** CT images demonstrated no residual mass compressing the spinal cord.

The postoperative examination reported no residual mass compressing the spinal cord (**Figure 6**). The weakness in both lower limbs improved immediately. However, the patient was hospitalized for five weeks because of complications. This patient developed cerebral fluid leakage within three days after the operation (Day 1:300ml, Day 2: 500ml and Day 3: 450ml) because of the inevitable incision of the dura during the operation. In addition, on the 4th postoperative day, the patient began to have an unexplained low-grade fever, so the patient was treated with antibiotics (ceftriaxone sodium, vancomycin, and meropenem). Finally, the patient was discharged five weeks after the operation with a normal temperature.

## 3 Discussion

According to PubMed, including the current case, altogether 34 articles (4–36) involving 43 cases have been published till 2022 (**Table 1**). This review indicated that the majority of OSM occur in women (female: 38, male: 5). The average age was 57.5 years (range from 15 to 90 years). Most patients with OSMs occurred in the thoracic spine (38 cases, 88.4%), followed by the cervical spine (4 cases, 9.3%) and lumbar spine (1, 2.3%). In terms of treatment, the tumors were removed in 38 cases by gross total resection (GTR), tumors in 3 cases were removed via subtotal resection (STR), and the extent of resection was not clearly described in the remaining 2 cases. The vast majority of patients improved gradually after the operation, and no recurrence (21 cases) was reported (the remaining not described).

**Table 1:** Summary of ossified meningioma cases.

Study (Year)	Age/Gender	Tumor number	Ossified	Level	Location	Symptoms	Treatment	Clinical outcomes	Recurrence	Histological characteristics
Roger et al, 1928 (4)	16/F	1	Ossified	T9	Lateral	Myelopathy	GTR	Improved (3 months)	No	Psammoma bodies, bone cells
Freidberg et al, 1972 (5)	69/F	1	Ossified	T1-2	Ventral	Myelopathy	GTR+dura	Improved (6 weeks)	NA	Psammoma bodies, mature cancellous bone
Kandel et al, 1989 (6)	17/F	1	Ossified	T8	Dorsal	Myelopathy	GTR	NA	No	Meningotheliomatous, psammoma bodies, bone spicule
Niijima et al, 1993 (7)	75/F	1	Ossified	T8-9	Dorsolateral	Myelopathy	GTR+dura	Improved (14 months)	NA	Psammoma bodies, bone spicule
Kitagawa et al, 1994 (8)	75/F	1	Ossified	T9-10	NA	Myelopathy	NA	NA	NA	Psammoma bodies, bone tissue
	60/F	1	Ossified	T6-8	NA	Myelopathy	NA	NA	NA	Psammoma bodies, bone tissue
Nakayama et al, 1996 (9)	74/F	1	Ossified	T9	Dorsal	Myelopathy	GTR	NA	NA	Matured lamellar bone tissue
	45/M	1	Ossified	C1-3	Ventral	Myelopathy	GTR	NA	NA	Matured bone tissue
Huang et al, 1999 (10)	73/F	1	Ossified	T5	Lateral	Myelopathy	GTR	Improved	NA	Psammoma bodies, bone marrow
Saito et al, 2001 (11)	54/F	1	Ossified	T11	Dorsal	NA	GTR+dura	Improved	No	Metaplastic (osseous)
Naderi et al, 2001 (12)	15/M	1	Ossified	T4	Dorsal	Myelopathy	GTR+dura	Improved (3 months)	NA	Psammoma bodies, mature bone tissue
Liu et al, 2006 (13)	70/F	1	Ossified	T11	Dorsolateral	Myelopathy	GTR	Improved (2 years)	No	Psammoma bodies, woven bone
Hirabayashi et al, 2009 (14)	82/F	1	Partially ossified	L3	Dorsolateral	Cauda equina syndrome	GTR	Improved	No (5years)	Osseous
Tahir et al, 2009 (15)	40/F	1	Partially ossified	T6	Dorsolateral	Myelopathy	GTR	Improved (8 months)	No	Mineralized bone
Uchida et al, 2009 (16)	40/F	2	Ossified	T8 and T12	Dorsal, dorsolateral	Myelopathy	GTR+dura	Improved	No (2years)	Psammoma bodies, mature bone
Licci et al, 2010 (17)	58/F	1	Ossified	T6	Dorsal	Myelopathy	GTR	Improved (1 year)	NA	Psammoma bodies, lamellar bone tissue, hematopoiesis
Chotai et al, 2013 (18)	61/F	1	Ossified	T4-5	Dorsal	Myelopathy	GTR+dura	Improved (1 month)	NA	Psammoma bodies, mature lamellar bone, hematopoiesis
Ju et al, 2013 (19)	61/F	1	Ossified	T9-10	Lateral	Myelopathy	GTR+dura	Improved (1 month)	NA	Heterotopic ossification
Taneoka et al, 2013 (20)	78/F	1	Ossified	T9	Dorsal	Myelopathy	GTR+dura	Improved	NA	Psammoma bodies, mature bone, hematopoiesis
Yamane et al, 2014 (21)	61/F	1	Ossified	T12	Ventrolateral	Myelopathy	GTR	Improved	No (2years)	Psammoma bodies, cancellous bone with bone marrow
Chan et al, 2014 (22)	64/F	1	Ossified	T9-10	Dorsal	Myelopathy	GTR	Improved (6 month)	NA	Psammoma bodies, bone marrow, hematopoiesis
Alafaci et al, 2015 (23)	45/M	1	Ossified	T2-3	Ventral 4, lateral 1, dorsal 4	Myelopathy	GTR	Improved	No	Seven cases of osseous component in association with psammoma bodies, two cases of immature bone trabeculae
	75/F	1	Ossified	T3-4		Myelopathy	GTR	Improved	No	
	86/F	1	Ossified	T3-4		Myelopathy	GTR	Improved	No	
	65/F	1	Ossified	T7		Myelopathy	GTR	Improved	No	
	72/F	1	Ossified	C7		Myelopathy	STR	Improved	No	
	40/F	1	Ossified	T1-2		Myelopathy	STR	Improved	No	
	65/F	1	Ossified	T7-8		Myelopathy	GTR	Improved	No	
	40/F	1	Ossified	C7		Myelopathy	GTR	Improved	No	
	41/F	1	Ossified	T2-3		Myelopathy	GTR	Improved	No	
Demir et al, 2016 (24)	26/F	1	Ossified-calcified	T9-11	Dorsal	Myelopathy	GTR	NA	NA	Psammoma bodies
Cochran et al, 2016 (25)	47/F	1	Ossified	T8	Ventral	Radiculopathy	GTR	Improved	No (22months)	Psammoma bodies, bone marrow, hematopoiesis
Xia and Tian, 2016 (26)	90/M	1	Ossified	T10-11	Dorsal	Spinal cord injury after fall	GTR	NA	NA	Psammoma bodies, bone trabeculae
Prakash et al, 2018 (27)	60/F	1	Ossified	T7-8	Dorsolateral	Myelopathy	GTR	Improved (6 month)	NA	Psammoma bodies, immature bony trabeculae
Sakamoto et al, 2018 (28)	57/F	1	Ossified	C7	Ventrolaterodorsal	Myelopathy	STR	Improved	NA	Osseous core, fibrous
Murakami et al, 2019 (29)	29/F	1	Ossified	T12	Lateral	Back pain, leg numbness	GTR+dura	Unchanged (12 months)	NA	Psammoma bodies, mature bone tissue
Taha et al, 2019 (30)	22/F	1	Ossified	T4-5	Dorsal	Myelopathy	GTR	Improved (6 month)	NA	Psammoma bodies, bone trabeculae
Wang et al, 2019 (31)	52/F	1	Ossified	T4	Dorsal	Back pain	GTR	Improved	No (2.5 years)	Psammoma bodies, immature trabecular bone, hematopoiesis
Xu et al, 2020 (32)	85/F	1	Ossified	T11	Lateral	Back pain, leg pain	GTR+dura	Improved	No (1 year)	Psammoma bodies
Buchanan et al, 2021 (33)	64/M	1	Ossified	T4	Dorsal	Myelopathy	GTR+dura	Improved (6 month)	NA	Psammoma bodies, bone formation, osseous metaplasia
Wong et al, 2021 (34)	75/F	1	Ossified	T10-T11	NA	Myelopathy	GTR+dura	Not improved (6 months)	NA	Psammoma bodies, immature trabeculae bone
Thakur et al, 2021 (35)	74/F	1	Ossified	T8	Ventrolateral	Tingling paresthesia	GTR+dura	Improved	NA	Psammoma bodies, bony hard-tissue fragments
Dong et al, 2022 (36)	76/F	5	Ossified	T7-12	Dorsal	Myelopathy	GTR+dura	Improved	No	Psammoma bodies, trabecular bone, hematopoiesis

F, female; M, male; T, thoracic; L, lumbar; C, cervical; GTR, gross total resection; STR, subtotal resection; NA, not available.

Spinal meningioma may originate from the arachnoid cap cells originating from the outer layer of the arachnoid mater and villi (18). According to the latest WHO classification, spinal meningioma can be divided into 15 different histological subtypes, with psammomatous, meningothelial and transitional being the most common histologic variants, but the calcification or ossification of spinal meningioma is uncommon (37). The specific pathogenesis of OSM remains controversial. One theory suggests that ossification is caused by the accumulation of hydroxyapatite crystals in psammoma bodies (38). However, some articles have reported that ossification can also occur without psammoma bodies, which may not support the theory (7, 19, 28). Therefore, most of the current reports believe that ossification begins with the metaplasia of arachnoid cells, which induces the synergistic effect of osteoblast, fibroblast, and angiogenesis components in bone tissue formation, thereby promoting ossification in spinal meningioma (7, 8, 19). Another study suggested that ossification of spinal meningioma can be induced by biochemical activation of the ossification cascade or by exposure to osteoblast transformation factors such as SOX9 and Runx-2 (16).

As demonstrated in **Table 1**, the incidence of OSM in females is significantly higher than in males. This phenomenon may be due to estrogen deficiency which increases the risk of calcification in regions containing necrotic fibroblasts (18). However, no evidence has been found that this phenomenon is related to sex hormones (39). In addition, the incidence of OSM is higher in the elderly, and OSM in children and young adults may not be as rare as in the general population.

The most OSMs have symptomatic but nonspecific clinical manifestations. The clinical features of these tumors include motor, sensory, and sphincter dysfunction, with different phenotypes depending on tumor location and nerve compression. Interestingly, all 43 cases of OSM demonstrated progressive myelopathy and worsened with time, but most patients did not report a history of symptoms within 2 years and some cases remained asymptomatic until events such as falls occurred. Depending on the peak age of onset, the mass considered asymptomatic or mild for a long time. The mass becomes unsteady after stimulation and may cause bleeding and displacement, which displace and cause nerve compression and worsen neurological symptoms.

In terms of diagnosis, OSM can sometimes be detected in a digital radiograph (DR), small calcification or ossification can be further observed in a CT scan, and the size or extent of OSM can be determined by MRI. However, since imaging cannot distinguish ossification or calcification, the ultimate diagnosis should be determined by histopathological findings (40). In addition, 18F-FDG PET/CT is not a routine examination for the diagnosis of OSM. However, recent studies have shown that new specific tracers, such as 68Ga-DOTA-SSTR and 68Ga-DOTATOC, can improve the diagnostic accuracy of meningiomas and assist radiosurgical or surgical planning (41, 42).

Once spinal meningioma is diagnosed, surgical resection is the primary treatment, which can relieve spinal cord compression, restore limb motor function, and reduce the pain of patients. Most intradural meningiomas are epiarachnoid masses, and the arachnoid mater becomes a barrier to separate the tumor from surrounding normal tissues during surgery. However, the unique differentiation form of OSM causes calcification or ossification of the arachnoid mater, which leads to the disappearance of this natural barrier. Moreover, compared with general spinal meningioma, OSM often sticks to the surrounding nerve tissue, which may be due to its scant cellularity consisting of acellular concretions (23). Therefore, patients with OSM often lack a clear anatomical view, which increases the risk and difficulties of surgery. At present, gross total resection (GTR) of OSM is considered to be most associated with significant neurological improvement. However, some researchers believe that when trying to remove a tumor with heavy adhesion may cause spinal cord injury, subtotal resection (STR) can be selected to retain part of the mass to ensure normal spinal cord function after surgery (43). With the development of medical technology, intraoperative neurophysiological monitoring (IONM) has been applied to determine the extent of resection during intradural extramedullary tumors (44). IONM can alert the operator to prevent spinal cord or nerve root injury by the condition of sustained electromyoelectric response. In conclusion, complete surgical removal of OSM is not easy, especially in OSM with complete ossification, but it remains the best choice available.

## 4 Conclusion

We presented a rare patient with a dorsal complete OSM in the thoracic spine. In the current report, only 1% to 5% of spinal meningiomas present with ossification, and the majority are in women. The diagnosis of OSM can usually be correctly diagnosed by observing the neuroradiological appearances. Surgical resection is the main therapeutic method for OSM, but it is somewhat challenging because tumor adhesion can prevent complete resection. In summary, treatment with GTR or STR often leads to clinical improvement with a low risk of late recurrence.

## Data availability statement

Please contact the corresponding author if data is required.

## Author contributions

W-BX: Conceptualization, Data curation, Methodology, Software, Visualization, Writing – original draft, Writing – review & editing. N-KS: Conceptualization, Supervision, Writing – review & editing. D-XC: Validation, Visualization, Writing – original draft. D-QC: Formal Analysis, Visualization, Writing – original draft. YN: Investigation, Writing – original draft. FJ: Validation, Writing – original draft. G-XL: Conceptualization, Funding acquisition, Writing – review & editing. GR: Conceptualization, Funding acquisition, Project administration, Resources, Supervision, Writing – review & editing.
